# Block-Wise Domain Adaptation for Workload Prediction from fNIRS Data

**DOI:** 10.3390/s25123593

**Published:** 2025-06-07

**Authors:** Jiyang Wang, Ayse Altay, Leanne Hirshfield, Senem Velipasalar

**Affiliations:** 1 Electrical Engineering and Computer Science Department, Syracuse University, Syracuse, NY 13244, USA; aaltay@syr.edu (A.A.); svelipas@syr.edu (S.V.); 2 Institute of Cognitive Science, University of Colorado Boulder, Boulder, CO 80309, USA; leanne.hirshfield@colorado.edu

**Keywords:** fNIRS, cognitive, workload, domain adaptation, contrastive learning

## Abstract

Functional near-infrared spectroscopy (fNIRS) is a non-intrusive way to measure cortical hemodynamic activity. Predicting cognitive workload from fNIRS data has taken on a diffuse set of methods. To be applicable in real-world settings, models are needed, which can perform well across different sessions as well as different subjects. However, most existing works assume that training and testing data come from the same subjects and/or cannot generalize well across never-before-seen subjects. Additional challenges imposed by fNIRS data include not only the high variations in inter-subject fNIRS data but also the variations in intra-subject data collected across different blocks of sessions. To address these challenges, we propose an effective method, referred to as the block-wise domain adaptation (BWise-DA), which explicitly minimizes intra-session variance as well by viewing different blocks from the same subject and same session as different domains. We minimize the intra-class domain discrepancy and maximize the inter-class domain discrepancy accordingly. In addition, we propose an MLPMixer-based model for workload prediction. Experimental results demonstrate that the proposed model provides better performance compared to three different baseline models on three publicly-available workload datasets. Two of the datasets are collected from *n*-back tasks and one of them is from finger-tapping. Moreover, the experimental results show that our proposed contrastive learning method can also be leveraged to improve the performance of the baseline models. We also present a visualization study showing that the models are paying attention to the right regions in the brain, which are known to be involved in the respective tasks.

## 1. Introduction

The level of cognitive workload (CWL) that a human experiences can affect their performance in human computer interaction (HCI) tasks. High levels of CWL can lead to human error, such as task shedding and frustration, while low CWL can cause boredom and complacency [1]. A real-world autonomous system that can dynamically adjust and assign tasks based on the CWL of different subjects can enhance both the efficiency and satisfaction of human participants. Such a system requires a CWL classification model that can generalize well to new subjects that have not been seen before during training.

Neuroscience researchers often use functional magnetic resonance imaging (fMRI) to study the brain activity of humans, since it has high spatial and temporal resolution [2]. However, fMRI is not very suitable for HCI research, since it is costly and prone to motion artifacts, and participants have to remain still while the data is collected. Therefore, HCI researchers have explored other methods to measure brain activity, such as electroencephalography (EEG) and functional Near-Infrared Spectroscopy (fNIRS). EEG measures the electrical potential on the scalp, which is generated by neural activation on the surface of the brain. It uses electrodes that are attached to the scalp. fNIRS, on the other hand, uses near-infrared light that can pass through the scalp and skull and reach the cortical surface. It measures the light that is reflected back from the brain tissue. The change in light intensity indicates the changes in oxy- and deoxy-hemoglobin concentration [3]. Some tasks and experiments may involve movement from participants, such as typing on a keyboard. fNIRS is more resistant to electrical noise and motion-based muscle activity artifacts than EEG [4].

In this work, we study the problem of classification of cognitive workload levels from fNIRS data. Methods have been proposed to employ deep learning for CWL classification. Yet, many approaches [5,6,7,8,9] train models per individual, and perform training and testing on the same subject. While these approaches have explored the potential of using deep learning on brain data, many challenges and issues still remain to be addressed. These issues include overfitting and inflated accuracy rates. When these models are tested on a new subject or even on the same subject during a different measurement session, the performance degrades significantly. This degradation may be in part due to the assumptions made by deep learning operations, such as training and testing data to have the same distribution, and the high reliance on Convolutional Neural Network (CNN)-based models [5,9,10].

The parameter sharing aspect [11] of CNNs is based on the assumption that a valid kernel of weights working for one position also works for other regions. For instance, in image classification, if there is a filter that can extract features of an object, then, no matter where the object is in the image, that filter will still be useful. Thanks to parameter sharing, CNNs have significantly less trainable parameters compared to multi-layer perceptrons (MLP). Although CNNs are considered to be invariant to small levels of transformations, later studies [12,13] have shown that this invariance assumption does not always hold. Moreover, there are some situations for which parameter sharing is not suitable. The prior fMRI literature on working memory load and selective attention has shown that the middle temporal gyrus is a region involved in the coordination between working memory and directed attention [14]. In addition, the human brain is highly interconnected, and some brain regions can work together in cognitive processing. Thus, the assumption of CNNs that a patch of weights learned from one spatial or temporal position can be used in another region does not always hold.

On the other hand, the number of probes in fNIRS devices and the corresponding fNIRS channels is usually less than 52 [4,15,16]. Hence, the processing of fNIRS data is not as computationally expensive as processing of images or videos. Motivated by these, instead of using CNNs, we propose an MLP-based method. More specifically, we employ an MLPMixer [17] architecture and adapt it to fNIRS data. MLPMixer is a general backbone originally proposed for the image classification task, wherein the input is a sequence of non-overlapping image patches. Each patch is projected to a desired hidden dimension as a token. Two types of MLP blocks are used in MLPMixer; one maps tokens along the sequence of tokens dimension, and another one maps tokens along the channel (feature depth) dimension. In contrast to this original approach, with fNIRS data, instead of dividing the input into patches, we project the entire spatial dimension to a token due to the limited resolution of brain signal data. Thus, in our version, the mixing happens along the temporal axis.

**Figure 1:**
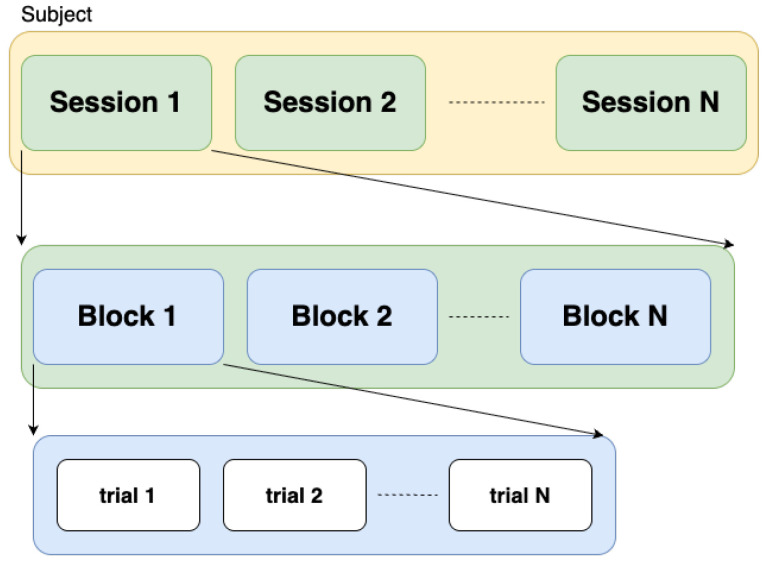
Commonly used experiment design when collecting fNIRS data. Each session is composed of a series of blocks, which are composed of a series of trials.

fNIRS data introduces additional challenges for autonomous processing tasks, including CWL classification. More specifically, the high variations in inter-subject fNIRS data (when subjects perform the same task) as well as in intra-subject data captured during different sessions need to be addressed. Block-wise experiments are a common method for collecting fNIRS data [4,16]. In this type of experiment, a participant performs a series of trials, which constitute a block, and a series of blocks constitutes a session. In each trial, participants are required to perform one specific task. Figure 1 illustrates the structure of block-wise experiments for one participant, where Session 1, Session 2, …, Session N denote different sessions.

The inter-subject variance arises due to the differences in type and style of hair, skin, skull, and brain structure. Intra-subject variances can occur due to differences in sensor placement across different sessions. Even within the same session, head motion, body motion, or noise due to the light source or electronics can cause difficulties [16,18]. To address these problems, researchers [16,18,19] have viewed data from different sessions of the same subject or data from different subjects as data from different domains, and used domain adaptation (DA) to align the data. In these prior works, some channels with low signal-to-noise ratios (SNRs) need to be removed before the alignment process. However, the dropping of channels can cause loss of useful information, and thus limit the performance, especially considering that the amount of fNIRS data and the number of channels is already limited to begin with. Zhong et al. [20] view different subjects as different domains to address the distribution difference between different subjects. In our earlier work [15], we presented a self-supervised method, which augments the data by arranging the controlled rest and task windows in different orders. This approach trains the deep learning model to classify the combinations of different orders and cognitive levels at the same time, and provides performance improvement on different types of cognitive tasks. However, the reliance on controlled rest data for training limits its application when no form of controlled rest signal is available in the data.

In order to address the aforementioned issues of high variance of inter-subject as well as intra-subject data for the same task, and eliminate the reliance on control rest data, we propose to view different blocks from the same/different subjects as different domains and use contrastive learning to align features extracted from same/different subjects for the same class. As mentioned above, previous works [19,20] treat data from different subjects or data from different sessions of the same subject as different domains and apply domain adaptation to align samples and help deep learning models generalize. However, as shown in Figure 1, fNIRS data is usually collected through multiple sessions, which are composed of a series of blocks, and variations can exist even for the same subject across different blocks. Thus, different from existing works, we propose a *block-aware loss* to also align samples from different blocks to improve the model generalization ability. The added benefit of this block-wise alignment is that it also helps align the features from different sessions. We present a detailed analysis and experimental results in Section 3.1 to motivate the use of our block-wise domain adaptation (BWise-DA) and demonstrate its benefits.

The main contributions of this paper include the following:

- We propose a contrastive learning method that aims to align the samples from different blocks within the same session, which belong to the same class. We also propose a block discrepancy term to measure differences between blocks.
- We propose a new classification model based on the MLPMixer by performing mixing along the temporal dimension.
- We perform a comprehensive set of experiments on three open-access datasets and show that our proposed classification model outperforms three different baselines. We also show the effectiveness of the block-wise domain adaptation on the workload classification performance.
- We show that our proposed block-wise contrastive learning module can be employed to improve the performance of other deep learning models for fNIRS data.
- We perform a brain area visualization study showing that models are paying attention to the right regions in the brain, which are known to be involved in the respective tasks.

A list of the key abbreviations used throughout this paper are provided in Table 1. The rest of this paper is organized as follows: Section 2 provides an overview of the related work on non-invasive brain data followed by a review of the recent deep learning methods used in HCI. The details of our proposed method are presented in Section 3. The description of the datasets and the details of training and testing splits are provided in Section 4 together with the experimental results and the related discussion. Ablation studies are presented in Section 5, and the paper is concluded in Section 6 with a summary and future work directions.

## 2. Related Work

Predicting workload (WL) has been of interest since as early as 1908 [21]. More recently, new approaches have been proposed to leverage machine learning for cognitive workload (CWL) classification. Several works [7,9] focus on per-participant model training due to high inter-subject as well as intra-subject cross-session variances existing in fNIRS data. Although providing good results, per-participant model training is not practical and not generalizable. As discussed above, more generalizable methods are needed for real-world applications, which can perform well on new data captured from never-before-seen participants. In this section, we first provide an overview of the work on non-invasive brain data, and summarize recent deep learning-based approaches that have been proposed to analyze brain signals.

### 2.1. Non-Invasive Brain Data

As mentioned above, although functional magnetic resonance imaging (fMRI) is very commonly used, thanks to its high spatial and temporal resolution [2], it has limitations as a research tool. In addition to being expensive, fMRI is very sensitive to motion artifacts. Participants need to keep still during data collection, since their movements can cause issues. For these reasons, researchers in the HCI domain have also focused on electroencephalography (EEG) [8,22,23] and functional Near-Infrared Spectroscopy (fNIRS) [8,9,15] data to measure brain activities. EEG and fNIRS data are spatio-temporal, i.e., these devices can capture data from multiple probes across the skull of participants, with specific layouts, and measure brain activity continuously in real-time.

A review of the history of fNIRS is provided in [24]. fNIRS provides higher spatial resolution than EEG, making it possible to better localize specific functional brain regions of activation, as could be done with the constrictive fMRI device [25]. Furthermore, the new frequency-domain (FD)-NIRS uses a linear symmetric dual-slope (DS) sensor design [26], which beneficially suppresses superficial hemodynamics, instrumental drifts, and motion artifacts [27].

Yet, the amount of publicly available fNIRS data has been limited, especially for studies covering a large number of participants. Available open access fNIRS datasets, such as TUBerlin [4] and FingerFootTapping (FFT) [28], usually contain data from 10–30 subjects. Tufts [16] is a more recent open access fNIRS dataset, which has been collected from 68 participants. In addition to containing data from a larger number of participants and representing a larger variation, the Tufts dataset also provides a standard evaluation protocol for machine learning training paradigms. This makes training and testing different neural networks and comparing them more convenient and commensurate.

### 2.2. Workload Classification with Deep Learning

While traditional machine learning (ML) algorithms, such as Support Vector Machines (SVMs), Linear Discriminant Analysis (LDA), Principal Component Analysis (PCA), k-nearest neighbors (kNN), and Artificial Neural Networks (ANN), have been widely used for mental workload classification, deep learning-based algorithms have become more popular in recent years, especially thanks to their ability of eliminating the need for handcrafted features.

Traditional ML algorithms, such as SVMs and LDA, have been employed for the detection of various levels of mental workload [29,30,31]. The main working principle of SVMs is finding a hyperplane that can separate the data accurately. If it results in a significantly smoother hyperplane in the optimization process, it provides a high generalization power by tolerating some misclassifications [32]. Compared to CNNs, SVMs are faster. However, SVMs employ handcrafted features and need selection of a best feature set. Their performance depends on the selection of these features and preprocessing. On the other hand, the main limitation of LDA in workload classification studies is its limited performance on nonlinear complex brain signals [33].

Schirrmeister et al. [23] proposed a deep convolutional model, referred to as the DeepConv, for EEG signals, and showed that end-to-end deep CNNs trained within-subject can provide promising accuracy. They first use 2D convolution (Conv2D) along the temporal dimension followed by convolution over the spatial dimension, and repeat this several times. At the end, they use a fully connected (FC) layer for final classification. EEGNet [22] also shows promising results on EEG signals across different experiments covering visual-, memory- and movement-related scenarios. EEGNet employs depth-wise separable convolution operators, which were introduced in the Xception architecture [34] for computer vision applications. EEGNet first uses Conv2D along the temporal dimension to learn frequency filters, and then employs depth-wise separable convolutions to combine frequency-specific features with spatial information for the final classifier, which is a single FC layer. Compared to DeepConv, EEGNet has far less trainable parameters. Saadati et al. [8] proposed a CNN architecture, for motor imagery and mental workload tasks, by using two types of brain data, namely fNIRS and EEG, together during training. The use of both modalities provides promising within-subject improvements compared to using single modality brain data.

Besides CNNs, there are other types of neural network architectures commonly used in deep learning. Recurrent neural networks (RNNs) are widely used especially in application areas concerned with sequential or temporal data, such as text, audio, and video [35]. Long Short Term Memory (LSTM) Networks [36,37] have been widely used to capture information along temporal dimension. Mughal et al. [9] proposed a CNN-LSTM architecture for mental workload classification within subjects. Unlike EEGNet and DeepConv, this type of architecture first uses a CNN to encode the spatial information, and then employs LSTM to capture the temporal information.

The aforementioned works mostly focus on within subject training. Cross-subject and cross-session CWL classification, on the other hand, is a much more challenging task due to the high variation in inter-subject fNIRS data and in intra-subject data across different sessions. Sommer et al. [38] use CNN-LSTM on classification of finger-tapping levels across sessions. In our earlier work [15], we replace the LSTM with a gated recurrent unit (GRU) [39], which has a similar structure to LSTM while having less trainable parameters. In [15], we propose to use self-supervised label augmentation [40] by permuting the order of data samples, as a [control rest-task pair] and [task-control rest] pair, and present promising results on multi-label classification of working memory load (WML) and visual perceptual load (VPL) across different subjects. Lyu et al. [19] view data from different sessions or from different subjects as data from different domains and use domain adaptation to align the data collected during *n*-back tasks. They show that this domain adaptation works on SVM, CNN, and RNN methods. In a more recent work, Wang et al. [41] use power spectral density (PSD) features to classify working memory load and employ a domain adaptation method to align features across different type of tasks and different subjects.

The aforementioned methods rely on CNNs to process spatial or temporal information. Yet, as discussed above, the assumption behind the parameter sharing aspect [11] of CNNs does not always hold for brain signals, since types and levels of workloads depend on specific brain areas [14] and some areas of brain are interconnected and work together in cognitive processing.

## 3. Proposed Method

In this work, we focus on the more challenging problem of cross-subject Cognitive Workload (CWL) and Motion Workload (MWL) classification from fNIRS signals. We propose an effective, end-to-end MLPMixer-based classifier with a contrastive learning data sampler, where the contrastive samples are collected both across different subjects and different sessions and blocks of the same and different subjects. The proposed contrastive learning approach is only employed during training and does not incur any additional computational cost during testing.

### 3.1. Motivation

As mentioned above, there are previous studies that treat different subjects [20] and sessions [19] as distinct domains and use domain adaptation (DA) to extract domain-invariant features and enhance the model’s generalization ability on unseen subjects or sessions. However, these works do not address or consider the fact that intra-subject variance can also arise within the same session across different blocks [16,18].

In this section, we perform *n*-back task classification to empirically show the performance of the DeepConv model [23] under different split scenarios in terms of Wasserstein loss [42] of the last layer of encoder and classification accuracy. Wasserstein loss [42] is widely used to measure the difference of two distributions. We evaluate the accuracy of DeepConv in four different split scenarios, namely split (i) by trial, (ii) by block, (iii) by session, and (iv) by subject, which are described as follows:

(i) Split by trial: involves using all the data from all subjects and sessions, and splitting the data for training and testing based on trials;
(ii) Split by block: involves using all the data from all subjects and sessions, and splitting the data for training and testing based on blocks;
(iii) Split by session: involves using the data from two (out of three) sessions of each subject for training, and the data from the remaining session of each subject for testing;
(iv) Split by subjects: involves using the data from a set of subjects for training, and the data from the remaining subjects for testing.

**Figure 2:**
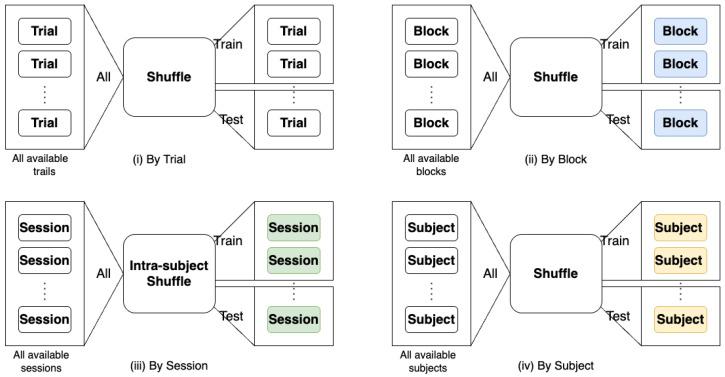
Four different split scenarios: Split by (**i**) trial, (**ii**) block, (**iii**) session, and (**iv**) subject.

These four split scenarios are illustrated in Figure 2. If the total dataset is envisioned as a multidimensional tensor comprised of four axes (trial, block, session, and subjects), for each scenario, we execute the data split along the corresponding axis, effectively dividing the dataset into distinct training and testing sets. This methodical partitioning ensures that our model training and evaluation processes are systematically structured allowing us to study effects of variations across trials, blocks, sessions, and subjects.

In this experiment, for each scenario, we measure how well DeepConv can classify the data from three-way *n*-back task (0-back vs. 1-back vs. 2-back) on the TUBerlin dataset. We use cross-entropy and train the model in a supervised way. Table 2 summarizes the results for different split scenarios. As can be seen, the DeepConv model achieves the highest accuracy of 51.42% when the data is split by trials. The accuracy decreases by 9.27%, when the data is split by blocks instead of trials. Similarly, the accuracy decreases by 9.07% when the data is split by sessions instead of trials, and by 8.62% when the data is split by subjects instead of trials. In summary, the highest accuracy is achieved when the data is split by trials, which can be explained by the fact that the model sees data from all subjects, sessions, and blocks during training in this split scenario. The lowest accuracy of 42.15% is obtained when the data is split by blocks, suggesting that the model cannot handle the intra-variance between blocks of the same task or condition.

We also employ the Wasserstein distance as a metric to quantify the discrepancy between the features of training and testing sets under each split scenario and report it as WD in Table 2. We measured the mean WD between different combinations of mini-batches from the training and testing data. As seen in Table 2, the lowest distance was achieved when the data was split by trials. This makes sense, since the data in each trial was collected in a short and continuous time span. This small WD is also in agreement with the highest accuracy obtained when data is split by trials. The highest WD was obtained when the data was split by sessions, while splits by subjects and by blocks resulted in similar distances.

The above sets of results show that the intra-subject difference between blocks should also be considered when developing new models for fNIRS data. The models’ accuracy is highly affected by the intra-subject variance of the data within each block of the same task or condition. This variability is as influential as the variability across new sessions, as well as new subjects. This is a significant point that has not been addressed in previous studies [16,18,19]. Motivated by these observations, we propose Block-Wise Domain Adaptation (BWise-DA) to explicitly decrease the difference between blocks in the same session of the same subject, and overcome the change of distribution between training and testing sets.

### 3.2. Preliminaries on Domain Adaptation

Domain adaptation (DA) aims to better generalize and improve the performance of models on target domain. In general, it is assumed that the distributions of the source domain samples $S=\{\left(x_{1}^{s},y_{1}^{s}\right),\cdots \left(x_{N_{s}}^{s},y_{N_{s}}^{s}\right)\}$ and target domain samples $T=\{\left(x_{1}^{t},y_{1}^{t}\right),\cdots \left(x_{N_{t}}^{t},y_{N_{t}}^{t}\right)\}$ are different, where $\left(x_{i}^{s},y_{i}^{s}\right)$ is a pair of input $x_{i}^{s}$ and its label $y_{i}^{s}$ from the source domain, and $\left(x_{i}^{t},y_{i}^{t}\right)$ is a pair of input $x_{i}^{t}$ and its label $y_{i}^{t}$ from the target domain.

To align the source and target domains, many DA methods use a feature extractor to create a representation that is invariant across domains. There are two common methods employed for this purpose. One approach is to minimize the distance between the features of the two distributions [43,44]. Another approach is to use a domain classifier in an adversarial way and reverse its gradient to make it fail to distinguish the domains [20,45]. Treating data from different subjects as different domains and mitigating the domain shift via DA can improve the cross-subject performance. This idea has been explored by Zhong et al. [20] for brain signal processing. To complicate matters further, with fNIRS data, we also face with high intra-subject variance in data captured from the same subject during different sessions, and even during the same session, as shown above. Hence, in this work, we propose to treat not only the data from different subjects but also different data blocks from the same subject as data from different domains.

### 3.3. Class-Aware Block-Aware Domain Adaptation

In this section, we first review the work on class-aware domain adaptation, referred to as the contrastive domain discrepancy (CDD) [44]. CDD is developed from Maximum Mean Discrepancy (MMD) [43]. In MMD, $\left\{x_{i}^{s}\right\}$ and $\left\{x_{i}^{t}\right\}$ are sampled from the marginal distributions $P(X^{s})$ and $Q(X^{t})$, respectively, which are independent and identically distributed (*iid*). MMD is motivated by the fact that if two distributions are identical, all statistics should be the same. MMD uses the mean embeddings in the reproducing kernel Hilbert space (RKHS) to describe the difference between two distributions. In practice, for the $l^{th}$ layer, $\phi_{l}$, of a neural network, the value of $D_{l}^{mmd}$ is estimated from the empirical kernel mean embeddings such that

(1) $$\begin{array}{ccc} D_{l}^{mmd} & = & \frac{1}{n_{s}^{2}}\sum_{i=1}^{n_{s}}\sum_{j=1}^{n_{s}}k_{l}\left(\phi_{l}\left(x_{i}^{s}\right),\phi_{l}\left(x_{j}^{s}\right)\right)\\ \; & + & \frac{1}{n_{t}^{2}}\sum_{i=1}^{n_{t}}\sum_{j=1}^{n_{t}}k_{l}\left(\phi_{l}\left(x_{i}^{t}\right),\phi_{l}\left(x_{j}^{t}\right)\right)\\ \; & - & \frac{2}{n_{s}^{2}n_{t}^{2}}\sum_{i=1}^{n_{s}}\sum_{j=1}^{n_{t}}k_{l}\left(\phi_{l}\left(x_{i}^{s}\right),\phi_{l}\left(x_{j}^{t}\right)\right), \end{array}$$

where $D_{l}^{mmd}$ represents MMD, $x^{s}\in S^{\prime }\subset S$, $x^{t}\in T^{\prime }\subset T$, and $S^{\prime }$ and $T^{\prime }$ are the mini-batch source and target data sampled from S and T, respectively. $k_{l}$ denotes the kernel selected for the $l^{th}$ layer of the neural network.

CDD is established on MMD. It explicitly incorporates the class information into the formula and measures intra-class and inter-class discrepancy across domains. Minimizing the intra-class domain discrepancy can cluster representations of samples within the same class, whereas maximizing the inter-class domain discrepancy pushes the representations from different classes far away from each other. CDD defines class-aware domain discrepancy as

(2) $$D_{c_{1}c_{2}}\left(\hat{y_{1}^{t}},\hat{y_{2}^{t}}\cdots \hat{y_{n_{t}}^{t}},\phi \right)=e_{1}+e_{2}-2e_{3},$$

where

(3) $$\begin{array}{ccc} e_{1} & = & \sum_{i=1}^{n_{s}}\sum_{j=1}^{n_{s}}\frac{\mu_{c_{1}c_{1}}\left(y_{i}^{s},y_{j}^{s}\right)k\left(\phi \left(x_{i}^{s}\right),\phi \left(x_{j}^{s}\right)\right)}{\sum_{i=1}^{n_{s}}\sum_{j=1}^{n_{s}}\mu_{c_{1}c_{1}}\left(y_{i}^{s},y_{j}^{s}\right)}\\ e_{2} & = & \sum_{i=1}^{n_{t}}\sum_{j=1}^{n_{t}}\frac{\mu_{c_{2}c_{2}}\left(y_{i}^{t},y_{j}^{t}\right)k\left(\phi \left(x_{i}^{t}\right),\phi \left(x_{j}^{t}\right)\right)}{\sum_{i=1}^{n_{t}}\sum_{j=1}^{n_{t}}\mu_{c_{2}c_{2}}\left(y_{i}^{t},y_{j}^{t}\right)}\\ e_{3} & = & \sum_{i=1}^{n_{s}}\sum_{j=1}^{n_{t}}\frac{\mu_{c_{1}c_{2}}\left(y_{i}^{s},y_{j}^{t}\right)k\left(\phi \left(x_{i}^{s}\right),\phi \left(x_{j}^{t}\right)\right)}{\sum_{i=1}^{n_{s}}\sum_{j=1}^{n_{t}}\mu_{c_{1}c_{2}}\left(y_{i}^{s},y_{j}^{t}\right)}. \end{array}$$

In Equation (2), c1 and c2 are classes to be used to calculate CCD. When c1=c2, it measures intra-class domain discrepancy; when $c_{1}\neq c_{2}$, it measures inter-class domain discrepancy. $\mu_{c_{1}c_{2}}$ is an indicator function such that

(4) $$\mu_{cc^{\prime }}\left(y,y^{\prime }\right)=\left\{\begin{array}{cc} 1 & if\;y=c,y^{\prime }=c^{\prime }\\ 0 & otherwise. \end{array}\right.$$

Finally, the contrastive domain discrepancy, $D^{cdd}$, can be calculated as

(5) $$\begin{array}{ccc} D^{cdd} & = & \frac{1}{M}\sum_{\begin{array}{c} c=1 \end{array}}^{M}D_{cc}\left(y_{1:n_{t}}^{t},\phi \right)\\ \; & - & \frac{1}{M(M-1)}\sum_{c=1}^{M}\sum_{\begin{array}{c} c^{\prime }=1\\ c^{\prime }\neq c \end{array}}^{M}D_{cc^{\prime }}\left(y_{1:n_{t}}^{t},\phi \right). \end{array}$$

Note that CDD [44] was proposed for unsupervised domain adaptation, i.e., $y_{1:n_{t}}^{t}$ is unknown and needs to be estimated by network module.

With fNIRS data, as will be discussed below, we not only adopt Class-Aware DA, but also view samples from different blocks of subjects as different domains. In our case, $y_{1:n_{t}}^{t}$ stands for the label of samples from one of the blocks of a subject in the training set.

### 3.4. Proposed Block-Wise Domain Adaptation

As discussed in Section 3.1, intra-subject variation across different blocks has a similar overall impact on the performance as different splits by sessions and subjects. To address this issue, we propose to explicitly take the block information into account and measure the intra-class variance across blocks from the same session of subjects. Intuitively, brain signals that share the same label from the same subjects ought to exhibit similar representations. We propose measuring the inter-block discrepancy by utilizing Maximum Mean Discrepancy (MMD) to evaluate the variations among blocks within the same session of a given subject.

(6) $$\begin{array}{ccc} D_{(z_{1},z_{2})|c}^{BWise} & = & \frac{1}{n_{z_{1}}^{2}}\sum_{i=1}^{n_{z_{1}}}\sum_{j=1}^{n_{z_{1}}}k_{l}\left(\phi_{l}\left(x_{i}^{z_{1}}\right),\phi_{l}\left(x_{j}^{z_{1}}\right)\right)\\ \; & + & \frac{1}{n_{z_{2}}^{2}}\sum_{i=1}^{n_{z_{2}}}\sum_{j=1}^{n_{z_{2}}}k_{l}\left(\phi_{l}\left(x_{i}^{z_{2}}\right),\phi_{l}\left(x_{j}^{z_{2}}\right)\right)\\ \; & - & \frac{2}{n_{z_{1}}^{2}n_{z_{2}}^{2}}\sum_{i=1}^{n_{z_{1}}}\sum_{j=1}^{n_{z_{2}}}k_{l}\left(\phi_{l}\left(x_{i}^{z_{1}}\right),\phi_{l}\left(x_{j}^{z_{2}}\right)\right) \end{array}$$

where $z_{1}|c$ and $z_{2}|c$ are samples from different blocks in the same class *c* of the same subject. Minimizing $D_{(z_{1},z_{2})|c}^{BWise}$ can decrease the difference between two conditional distributions $P(X_{z_{1}}|Y)$ and $P(X_{z_{2}}|Y)$. In other words, during training, a pair of data with the same label from the different blocks of the same session of the same subject will be the input and output the MMD.

### 3.5. The Architecture of the Modified MLPMixer

MLPMixer [17] is fully built upon MLP layers. As mentioned above, workload is related to brain areas, which are usually interconnected. CNNs assume that a patch of weights valid for one position is also useful for other locations, which does not always hold for fNIRS data. Thus, the fully connected nature of MLPs is more suitable for analyzing fNIRS data compared to CNNs. Moreover, fNIRS data is relatively smaller compared to images and videos, making the use of MLP layers more feasible in this case.

**Figure 3:**
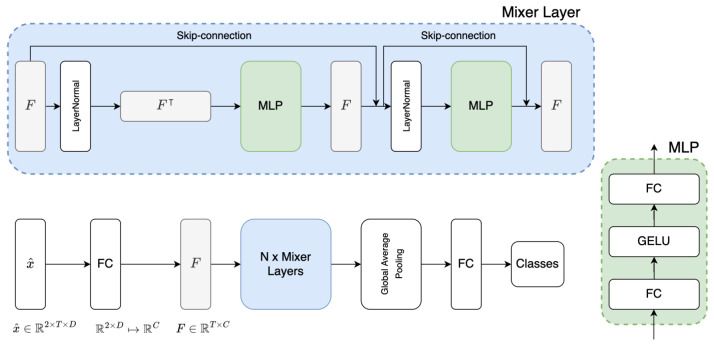
The MLPMixer-based classifier adapted for fNIRS data. The input data $\hat{x}\in R^{2\times T\times D}$ is first encoded by an FC layer ($R^{2\times D}\mapsto R^{C}$), and then follows the steps in the original MLPMixer [17]: the encoded feature (*F*) is sent to *N* mixer layers. Each mixer layer contains one temporal-mixing MLP ( $R^{T}\mapsto R^{T}$) and one channel-mixing MLP ( $R^{C}\mapsto R^{C}$). An MLP has two FC layers and a GELU nonlinearity. Other components include layer normalization, skip-connection, and global average pooling.

MLPMixer [17] was proposed as an image classification model. It takes a sequence of non-overlapping image patches as the input, and projects each patch to a desired hidden dimension as a token $F\in R^{S\times C}$, where *S* is the length of input sequence, and *C* is the size of the hidden dimension of token. MLPMixer contains multiple mixer layers, and each mixer layer consists of two MLP blocks. The first MLP block is a token-mixing MLP, which is applied to the columns of input (i.e., $F^{⊺}$), and maps $R^{S}\mapsto R^{S}$. The second MLP block is the channel-mixing MLP, which is applied to the rows of input *F*, and maps $R^{C}\mapsto R^{C}$. MLPMixer is a general backbone network for the image classification task. In this work, we modify the MLPMixer for the fNIRS classification task. The Tufts dataset we use has data from two fNIRS channels (i.e., $\hat{x}\in R^{2\times T\times D}$). Thus, we add a fully connected (FC) layer ahead of MLPMixer, mapping $R^{2\times D}\mapsto R^{C}$, which corresponds to the token-mixing of the original MLPMixer. Figure 3 shows the architecture of the MLPMixer we use. In our experiments, the parameters of the MLPMixer are set as follows: T=150, D=4, C=16, N=4. For the temporal-mixing MLP, the hidden dimension is 64. For the channel-mixing MLP, the hidden dimension is 32. For the TUBerlin and FFT datasets, which have different numbers of fNIRS channels compared with the Tufts dataset, we adjust the FC layer accordingly.

An MLP block can be written as follows:

(7) $$F=w_{2}\sigma \left(w_{1}\left(x\right)+b_{1}\right)+b_{2},$$

where σ is an element-wise non-linearity GELU [46] layer and $w_{1}$ and $b_{1}$ are the weights and bias, respectively, for the first FC layer. They map the input feature to a hidden feature. More specifically, $R^{T}\mapsto R^{T_{h}}$ with temporal-mixing and $R^{C}\mapsto R^{C_{h}}$ with channel-mixing, where $T_{h}$ and $C_{h}$ are the hidden dimensions for temporal-mixing and channel-mixing, respectively. Similarly, $w_{2}$ and $b_{2}$ are the weights and bias, respectively, for the second FC layer mapping the hidden feature to the same dimension as the input feature. More specifically, $R^{T_{h}}\mapsto R^{T}$ with temporal-mixing and $R^{C_{h}}\mapsto R^{C}$ with channel-mixing. The output feature of an MLP block has the same dimension as the input feature to fit the residual skip-connection, which is a commonly used deep-learning approach proposed in ResNet [47].

### 3.6. The Overall Objective

The overall objective can be written as

(8) $$\min_{\theta }l=l^{ce}+\alpha \left(D^{cdd}+D^{BWise}\right),$$

where θ represents the training parameters of the network, $l^{ce}$ is the cross-entropy loss, and α is the weight for the discrepancy penalty term. The minimization of the cross-entropy loss is aimed at supervised classification tasks. Minimizing $D^{cdd}$ aims to reduce the intra-class discrepancy while enhancing the inter-class discrepancy. Minimizing $D^{BWise}$ allows for minimizing the intra-subject difference. All three terms contribute to decreasing the performance drop, caused by domain shift and session inconsistency, when tested on brain signals of never-before-seen subjects.

## 4. Experiments

In this section, we present the results of our experiments performed on three publicly available and commonly used fNIRS datasets. After first introducing these datasets, we compare the performance of our proposed method with three different baselines, namely DeepConv [23], EEGNet [22], and MLPBiGRU [15], on these datasets, and then present the visualization results.

### 4.1. Datasets and the Experiment Setup

We conduct our experiments on the TUBerlin [4], Tufts [16], and FingerFootTapping (FFT) [28] datasets. The TUBerlin [4] and Tufts [16] datasets were collected from *n*-back tasks, and the FignerFootTapping (FFT) [28] dataset was collected when participants were performing finger- and foot-tapping tasks.

TUBerlin Dataset: This dataset, presented by Shin et al. [4], contains simultaneous EEG and fNIRS recordings of 26 participants, who performed three kinds of cognitive tasks: *n*-back, discrimination/selection response task (DSR), and word generation (WG) tasks. The dataset is open-access and multi-modal, capturing brain activity from different sources. We use the data from the *n*-back task to evaluate our method. The *n*-back task involves 0-, 2-, and 3-back levels, and our aim is to classify the mental workload levels across subjects according to the difficulty of *n*-back tasks. As *n* increases, the working memory workload increases. The dataset of the *n*-back task consists of three sessions. In each session, the subjects completed three blocks of 0-, 2-, and 3-back tasks in a counterbalanced order (i.e., 0 → 2 → 3 → 2 → 3 → 0 → 3 → 0 → 2). Thus, there are a total of nine blocks of *n*-back tasks for each subject. Each block starts with a 2-s instruction that shows the task type (0-, 2-, or 3-back), followed by a 40-s task period, and ends with a 20-s rest period. During the task period, a single-digit number was randomly presented every 2 s, resulting in twenty trials per block. For each *n*-back task, there are 180 trials in total (20 trials × 3 blocks × 3 sessions).

fNIRS data is acquired by a NIRScout device (NIRx Medizintechnik GmbH, Berlin, Germany) and further converted to deoxy-(HbR) and oxy-hemoglobin (HbO) intensity changes, using modified Beer-Lambert law [48], and downsampled to 10 Hz. Following downsampling, a low-pass filter (6th order zero-phase Butterworth) with a 0.2 Hz cut-off frequency was applied to remove high-frequency instrument noise and systemic physiological artifacts, instead of using a band-pass filter.

The fNIRS device has sixteen sources and sixteen detectors that were positioned on the frontal, motor, parietal, and occipital regions of the head. An fNIRS channel is formed by a source-detector pair that was next to each other, resulting in 36 channels in total. Each channel has two features corresponding to the ΔHbR and ΔHbO data. The total number of features is 72, i.e., 36 spatial locations × 2 hemoglobin types × 1 optical data type (intensity).

FFT Dataset: Bak et al. [28] presented an open-access fNIRS dataset, which contains data from 30 participants for three-class classification, namely left-hand unilateral complex finger-tapping (LHT) (Class 0), right-hand unilateral complex finger-tapping (RHT) (Class 1), and foot-tapping (FT) (Class 2). In each session, the order of tasks is randomly generated. A trial starts with a 2-s introduction, and then 10 s of task period followed by an 17–19 s of inter-trial break. There are a total of 225 trials (25 trials × 3 task types × 3 sessions).

fNIRS data was recorded by a three-wavelength continuous-time multi-channel fNIRS system (LIGHTNIRS, Shimadzu, Kyoto, Japan) consisting of eight sources and eight detectors. Four of the sources and detectors were placed around C3 on the left hemisphere, and the rest were placed around C4 on the right hemisphere. The raw fNRIS data was further converted to the intensity changes i.e., ΔHbR and ΔHbO using modified Beer-Lambert law [48] with a sample rate of 13.33 Hz. Then, data was band-pass filtered through a zero-order filter implemented by a third-order Butterworth filter with a pass-band of 0.01–0.1 Hz to remove physiological noises. There are 20 fNIRS channels and the total number of features is 40, i.e., 20 spatial locations (10 for each hemisphere) × 2 hemoglobin types × 1 optical data type (intensity).

Tufts Dataset: More recently, Huang et al. [16] presented the largest open-access fNIRS dataset including data from 68 participants performing *n*-back tasks. The *n*-back tasks involve 0-, 1-, 2-, and 3-back levels, and our aim is binary classification between 0- and 2- levels, same as what was achieved in Huang et al. [16]. Each subject performed tasks in only one session, and completed 16 blocks of *n*-back trials in a counterbalanced order (i.e., 0 → 1 → 2 → 3 → 1 → 2 → 3 → 0 → 2 → 3 → 0 → 1 → 3 → 0 → 1 → 2). Each block contains 40 trials lasting a total of 80 s (each trial lasting 2 s), followed by 10–30 s of rest period. For each participant, there are 640 trials in total (40 trials × 16 blocks × 1 session).

fNIRS data is acquired by an Imagent frequency-domain (FD) NIRS instrument manufactured by ISS (Champaign, IL, USA). Two sets (left and right) of custom probes with linear symmetric dual-slope (DS) [26] sensor design were placed on the forehead. The raw data was further converted to the changes of HbR and HbO in intensity and phase [27] and sampled at 10 Hz. Then, each univariate time-series was band-pass filtered using a third-order zero-phase Butterworth filter, retaining 0.001–0.2 Hz to remove noise. The data consists of total of eight features, coming from 2 spatial locations × 2 hemoglobin types × 2 optical data types (intensity and phase).

Experiment Setup: We used the data from all three datasets as is, without further preprocessing, since data had already been filtered with band-pass/low-pass filters to eliminate noise. We created the input data with sliding windows. The window size was the same as the task period of each trial, i.e., 2 s for TUBerlin and 10 s for FFT. For the Tufts dataset, we used a 15-s window as recommended by the original paper. The input shapes and corresponding sliding window durations that we used in our experiments, for all three datasets, are listed in Table 3. It should be noted that, in FFT, the experiments are not in block-wise design. Thus, for FFT dataset, we applied our proposed BWise-DA on sessions instead of blocks.

All models, including our proposed method and the baseline models, were trained from scratch on a server equipped with a single NVIDIA RTX 2080 GPU (NVIDIA, Santa Clara, CA, USA), running Ubuntu 22.04 with Python 3.9 and PyTorch 1.12. Training was stopped when the evaluation loss did not improve for 50 consecutive epochs. We used the Adam optimizer [49] for training. We performed a grid search to find the best hyper-parameters, namely the learning rate and the dropout ratio for all models. The learning rate was chosen from {$1\times 10^{-4},1\times 10^{-3},1\times 10^{-2},1\times 10^{-1}$} and the dropout ratio was chosen from {0, 0.25, 0.5, 0.75}. The value of α in Equation (8) is selected by grid search from {0.5, 0.6, 0.7, 0.8, 0.9, 1.0, 1.1} based on the test mean accuracy of the MLPMixer model. The learning rate was $1\times 10^{-3}$, the dropout ratio was 0.0, and the α was set as 1.1 for Tufts, and as 1.0 for TUBerlin and FFT datasets.

### 4.2. Experimental Results

We have conducted four sets of experiments on three open-access datasets and compared our MLPMixer model with three baselines, namely DeepConv, EEGNet, and MLPBiGRU. We used *k*-fold cross-subject validation and reported the mean accuracy and the F1 score over *k* folds. For both TUBerlin and FFT datasets, we split the data into 10 folds based on subject IDs. In the TUBerlin dataset, each fold contains 18 subjects for training, 5 subjects for validation, and 3 subjects for testing. In the FFT dataset, each fold consists of 21 subjects for training, 6 subjects for validation, and 3 subjects for testing. For Tufts dataset, we used the splits provided in the original paper [16], which also separated the folds by participants. Experimental results obtained on TUBerlin, FFT, and Tufts datasets, without DA and with different DA scenarios (subject-, session-, and block-wise), are shown in Table 4, Table 5 and Table 6, respectively. In our presentation of the results, we highlight the highest value in each column in bold. The overall highest value in the entire table is both bolded and underlined for clear distinction. For domain adaptation (DA) methods, the differences between the DA outcomes and those obtained through training with cross-entropy (CE) are displayed in parentheses. A green color indicates an increase in performance thanks to the DA method, while a red color signifies a decrease.

In the first set of experiments, we only used cross-entropy (CE in tables), without domain adaptation, to train our proposed MLPMixer and compare its performance as a backbone with three different baselines. Table 4, Table 5 and Table 6 show that, without DA, our proposed MLPMixer provided a mean accuracy of 41.47% (Table 4) on the three-way workload *n*-back (0- vs. 2- vs. 3-back) classification task on TUBerlin dataset, which is 1.56% lower than the best accuracy obtained by EEGNet. The classification task for TUBerlin is a three-way classification, in contrast to the binary classification with the FFT and Tufts datasets, making the task on this dataset more challenging. In addition, each sample from the TUBerlin dataset captures only 2 s of brain activity, a considerably shorter duration than the 10 s samples from the FFT dataset and the 15 s samples from the Tufts dataset. Moreover TUBerlin dataset contains the smallest number of subjects, with only 26 participants, compared to 30 and 68 participants in the FFT and Tufts datasets, respectively. The combination of these factors hinders the ability to demonstrate and highlight the generalizability of MLPMixer across a large number of subjects. Despite these challenges, the MLPMixer combined with our proposed Blockwise-DA achieves the highest F1 score on this dataset. On the FFT dataset (from 30 participants), our MLPMixer provided 64.76% (Table 5) accuracy on the three-way LHT vs. RHT vs. FT classification task, outperforming all three baselines. For the binary workload *n*-back (0- vs. 2-back) classification task on the Tufts dataset (from 68 participants), our MLPMixer again provided the highest mean accuracy of 67.28% (Table 6), which is 0.95% higher than the second best accuracy obtained by MLPBiGRU. From these results, we can see that our proposed MLPMixer model shows better generalizability, even before DA, in handling fNIRS data obtained in different settings and for different tasks across subjects.

In the second sets of experiments, we assessed the impact of our proposed block-wise domain adaptation approach on our proposed MLPMixer model as well as on three baseline models. The results on TUBerlin dataset (last two columns of Table 4) show that our proposed BWise-DA approach improves the F1 score of all models. The most significant improvement was achieved by applying BWise-DA to our MLPMixer-based model, which improved the accuracy from 41.47% to 42.78%, providing an increase of 1.31%, and improved the F1 score from 0.3899 to 0.4112. As for the FFT and Tufts datasets, our proposed BWise-DA approach improved the accuracy and F1 scores of *all models*. Accuracy improvements of as much as 2.76% and 4.43% are achieved on the FFT and Tufts datasets, respectively, thanks to our block-wise DA approach. For the FFT dataset (last two columns of Table 5), MLPBiGRU model experienced the largest improvement by using BWise-DA. More specifically, our block-wise DA approach improved its accuracy from 60.62% to 63.38%, providing an increase of 2.76%, and improved the F1 score from 0.5775 to 0.6279. With the incorporation of BWise-DA, our proposed MLPMixer provided the best overall performance on both FFT and Tufts datasets.

To explore the effect of treating samples from different blocks as different domains versus treating different subjects and sessions as different domains, we conducted two additional sets of experiments. When we treat different subjects as different domains, the second term of Equation (8), i.e., $D^{BWise}$, reduces to zero. When we treat different sessions as different domains, then $D^{BWise}$ measures the discrepancy between samples with the same label from different sessions of the same subject. The results for subject-wise and session-wise DA are presented in the respective columns of Table 4, Table 5 and Table 6. Since there is only one session for each subject in the Tufts dataset, the Session DA column of Table 6 is not applicable. In all 12 cases, our proposed BWise-DA (with block wise DA) outperforms Subject-DA and Session-DA on TUBerlin and FFT datasets. For the Tufts dataset, BWise-DA outperforms Subject-DA for all models. As for the comparison of Subject-DA versus Session-DA, for five out of eight comparisons on TUBerlin and FFT datasets, Session-DA outperformed Subject-DA. Overall, for all 12 comparisons, our proposed BWise-DA provides improvement in the F1 score over CE (not using domain adaptation) and for 11 out of 12 comparisons, our proposed BWise-DA provides accuracy improvement over CE. In addition, all the overall best performances are reached by incorporating BWise-DA for all three datasets.

Our proposed MLPMixer with BWise-DA provided the highest F1 score on all three datasets. Paired *t*-tests across Blockwise-DA and CE (Table 7) revealed significant F1 score improvements (*p* < 0.05) for MLPBiGRU on the FFT and for MLPMixer on TUBerlin dataset. On the Tufts dataset, Blockwise-DA provided significant improvement for three of the four models.

These results across three open-access fNIRS datasets demonstrate that our MLPMixer-based model has a high generalizability on fNIRS data for different types of tasks, such as three-way or binary *n*-back classification and the LHT vs. RHT vs. FT motion workload classification task. We also show that our proposed BWise-DA approach is versatile and effective when used with different kinds of networks, such as CNN-based (DeepConv and EEGNet), RNN-based (MLPBiGRU), and MLP-based (MLPMixer) methods, for the cognitive workload classification task. Moreover, we observe similar improvements on motion workload classification task.

In terms of computational complexity, our method does not add extra modules to networks. However, compared to conventional training with cross-entropy, training with BWise-DA incurs additional computational effort due to the calculation of $D^{cdd}$ and $D^{BWise}$, necessitating the pairing of samples for network training. Given the shallow and lightweight nature of these networks, the increase in computational demand is minimal, which can be easily handled by modern computers equipped with graphics cards. More importantly, at the inference stage, BWise-DA does not introduce any extra computational cost. In our comparison of the Multiply-Accumulate operations (MACs) between baseline models and our proposed MLPMixer model across three datasets at inference, as detailed in Table 8, we observe that our MLPMixer model ranks as the second least computationally intensive (after EEGNet) among the evaluated models. With the incorporation of BWise-DA, our MLPMixer model attained the highest accuracy on both the FFT and Tufts datasets. The compactness of EEGNet, being the smallest model evaluated, minimizes the risk of overfitting, especially for smaller datasets. Specifically, TUBerlin dataset contains data from only 22 participants, compared to the larger Tufts dataset, which comprises data from 68 participants. As seen by comparing Table 4, Table 5 and Table 6, EEGNet’s performance, in comparison to other models, degrades on larger datasets, on which larger models can be better trained.

### 4.3. Visualization of Brain Regions

In this section, we visualize the brain regions, which are most influential on the workload classification outcome, by measuring how the performance changes when different fNIRS channels are masked, i.e., thrown away. To determine the most important areas for brain–computer interface (BCI) performance, we used two datasets with high-density fNIRS channels, namely TUBerlin and FFT datasets. We did not use the Tufts dataset, which is collected by only two probes on the frontal cortex.

**Figure 4:**
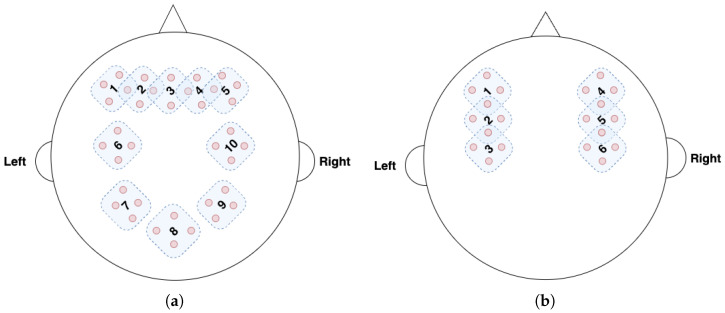
The fNIRS channels and masking positions for (**a**) TUBerlin and (**b**) FFT datasets. Red dots represent the fNIRS channels and mask positions are numbered. Each mask covers 4 channels. There are 36 channels and 10 masks for the TUBerlin dataset, and 20 channels and 6 masks for the FFT dataset.

Figure 4a and Figure 4b show the fNIRS channels for the TUBerlin and FFT datasets, respectively, with the numbered masks. We used MLPBiGRU-BWise-DA and MLPMixer-BWise-DA on the TUBerlin and FFT datasets, respectively, since these combinations provided the best performance on the respective datasets. We applied each mask one at a time, i.e., we removed the data from the masked channels, and measured the change in accuracy of the trained model. More specifically, for each mask, we set corresponding channel values to 0 to block out the information contained at that position.

**Figure 5:**
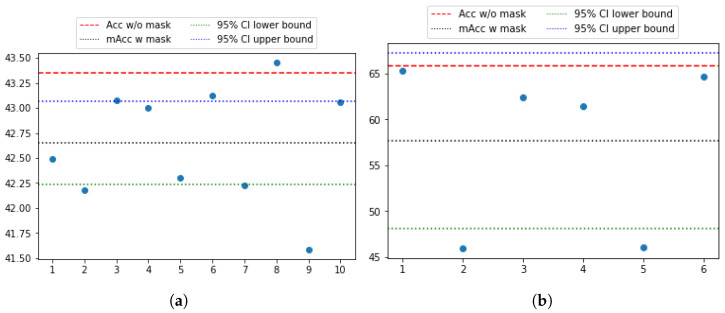
Accuracy plot when using one mask (discarding four channels) at a time with (**a**) MLPBiGRU-Block-DA on TUBerlin and (**b**) MLPMixer-Block-DA on FFT. The x-axis shows the mask number and the y-axis shows the k-fold cross validation accuracy on test splits. The black dotted line represents the average performance across all remaining channel positions. The red dash line shows the performance with no masking. The green and blue dot-dash lines indicate the lower and upper bounds of the 95% confidence intervals of the masked accuracy.

Figure 5 shows the results of this masking experiment. The black dotted lines indicate the average accuracy over all the remaining channels after masking for each dataset. The channels that caused a significant drop in accuracy, below the lower bound of the 95% confidence interval (green dotted line), were considered critical channels. As seen in Figure 5a, for the TUBerlin dataset, the critical channels are 2, 7, and 9, which correspond to the AF3, P3, and P4 areas of the brain [4,50], respectively. The AF3 area is located in the left of the midline of the prefrontal cortex, which is considered as a part of working memory load. The P3 area is located in the left parietal lobe. Mirroring P3, P4 is located over the right parietal lobe. Our results indicate that areas in the prefrontal cortex and parietal lobes are involved when solving the *n*-back task. For the FFT dataset, mask positions 2 and 5 (Figure 5b) result in lower accuracy than the 95% confidence interval lower bound. These mask positions are associated with brain areas C3 and C4, respectively [28], which are situated within the primary motor cortex. Hence, our findings are consistent with these brain areas, since the FFT dataset was collected when participants were performing motor tasks of finger- or foot-tapping. Results demonstrate that the model is focusing on the right brain areas, since the C3 and C4 regions are predominantly associated with the processing of motion-related workload.

## 5. Ablation Studies

### 5.1. Effectiveness of the Block-Wise Discrepancy Term, $D^{BWise}$

The effect of the $D^{BWise}$ term, and using block-wise domain adaptation was reported in Table 4, Table 5 and Table 6, on the TUBerlin, FFT, and Tufts datasets, respectively, by comparing its performance with different DA scenarios, namely subject-wise DA and session-wise DA. As discussed above, in all 12 comparison experiments, our proposed BlockWise-DA outperforms Subject-DA and Session-DA on the TUBerlin and FFT datasets. For the Tufts dataset, BWise-DA outperforms Subject-DA for all models. In summary, the overall best performances are reached by incorporating BWise-DA for all three datasets.

### 5.2. Role of α

**Figure 6:**
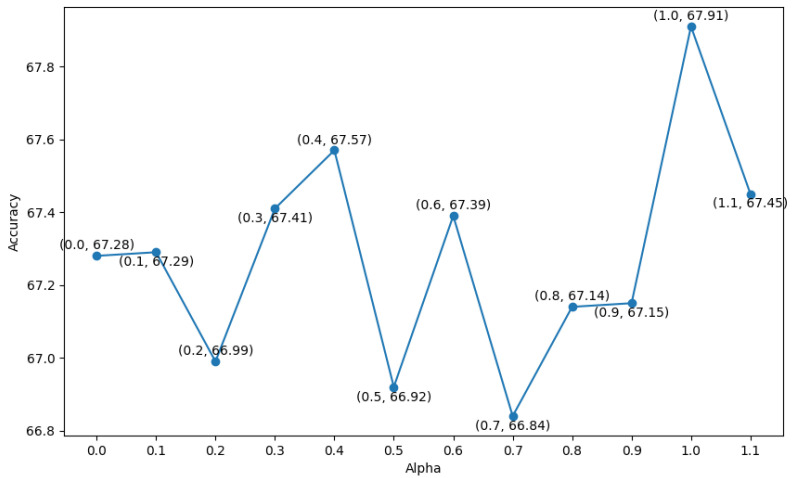
Mean accuracy (y-axis) of our MLPMixer for 17-fold cross-validation across participants on Tufts dataset against different values of α on the x-axis.

We also trained our model using BWise-DA and with different values of α. As seen in Equation (8), α weights the $D^{BWise}$ and $D^{cdd}$ terms in the objective function. We conduct our experiments on the Tufts dataset, since it has the largest amount of participants and the original paper [16] has released 17-fold splitting. We report the grid search of α starting from 0.0 to 1.1 with step size of 0.1. The mean accuracy of our MLPMixer is reported in Figure 6, which shows how the mean accuracy of MLPMixer varies with different values of α.

The mean accuracy is calculated by averaging the accuracy of 17-fold cross-validation across participants. The plot reveals that the highest mean accuracy of 67.91% is achieved when the α is set to 1.0.

## 6. Conclusions

One of the major challenges with CWL classification in real-world scenarios is the high variability of fNIRS data across different subjects and even across the same subject in different sessions. This high intra-class and intra-subject variance demands a model that can generalize well to never-before-seen participants. Most of the existing works have adopted a per-participant approach for model training. Some more recent works have employed domain adaptation (DA) to reduce the high variability by treating different subjects or sessions as different domains. We have demonstrated that the variability for the same subject across different blocks is also significant, by comparing the accuracy of different splitting methods for the same dataset. We have also computed the Wassertein distance to measure the level of difference between training and testing with different splitting methods, and found that splitting by blocks has similar level of difference as splitting by subjects. To alleviate the influence of intra-subject variance and improve the model generalizability, we have proposed to view blocks as different domains. To achieve this, we have proposed a Block-Wise (BWise) domain adaptation term to explicitly measure the difference of the distributions of different blocks within the same session of the same subject. We have also discussed the limitation of the CNN models that assume spatial invariance, which is not suitable for fNRIS data. We have proposed to use an MLPMixer-based approach instead of CNNs. Our experimental results have shown that MLPMixer as the baseline model (trained only with cross entropy) has achieved a significant improvement on the FFT and Tufts datasets. Our proposed BWise-DA has consistently improved the performance of all four models on the Tufts dataset for *n*-back tasks, and on the FFT dataset for finger- and foot-tapping tasks. It has also enhanced the performance of three out of four models on the TUBerlin dataset for *n*-back tasks. We have also performed a visualization study showing that the model is paying attention to the right regions in the brain, which are known to be involved in the respective tasks. Our ablation studies also have shown the effectiveness of the block-wise domain adaptation term in the overall objective function. Our method introduces an additional hyper-parameter to balance the cross-entropy loss with the new block-wise domain adaptation term. Optimal performance hinges on careful hyper-parameter selection. Specifically, in the paired *t*-tests, Blockwise-DA provided statistically significant improvement (*p* < 0.05) for different models on different datasets (not for all 12 comparisons). For Blockwise-DA, a small block size limits the number of samples available from the source domain. This scarcity increases the risk of overfitting, which can ultimately degrade final performance. Moving forward, future work will focus on refining the domain adaptation method to enhance stability and eliminate the need for this newly introduced hyper-parameter. We also plan to extend our work beyond fNIRS data to other brain data modalities, such as EEG, which share similar properties with fNIRS.

By addressing the intra-subject variability through our Block-wise domain adaptation approach, we enhance the reliability and generalizability of fNIRS-based models. This improvement has the potential to facilitate more accurate and consistent monitoring of brain function and workload across different subject populations, including never-before-seen subjects, in real-world settings including clinical ones.

## Figures and Tables

**Table 1:** Table of key abbreviations, ordered by their first appearance.

fNIRS	Functional Near-Infrared Spectroscopy	MMD	maximum Mean Discrepancy
fMRI	Functional Magnetic Resonance Imaging	RKHS	Reproducing Kernel Hilbert Space
EEG	Electroencephalography	iid	Independent and Identically Distributed
BWise-DA	Block-wise Domain Adaptation	GELU	Gaussian Error Linear Unit
DA	Domain Adaptation	CE	Cross-Entropy
SNR	Signal-to-Noise Ratio	FFT	FingerFootTapping (dataset)
WML	Working Memory Load	LHT	Left-Hand Unilateral Complex Finger-Tapping
VPL	Visual Perceptual Load	RHT	Right-Hand Unilateral Complex Finger-Tapping
WD	Wasserstein Distance	FT	Foot-Tapping
CDD	Contrastive Domain Discrepancy	MACs	Multiply-Accumulate operations

**Table 2:** Performance of DeepConv on TUBerlin dataset for *n*-back classification task (0-, vs. 2- vs. 3-back) at different split scenarios. “WD” and “Acc” represent Wasserstein Distance and accuracy, respectively.

Split-By-		Subject	Session	Block	Trial
DeepConv	WD	4.16 × 10^−3^	5.65 × 10^−3^	4.83 × 10^−3^	3.20 × 10^−3^
	Acc	42.80%	42.35%	42.15%	51.42%

**Table 3:** Input shape and sliding window duration for three datasets. Input sample $x\in R^{S\times D}$, where *S* and *D* represent sequence length and number of features, respectively.

Dataset	Input Shape	Duration
TUBerlin	$x\in R^{20\times 72}$	2 s
FFT	$x\in R^{134\times 40}$	10 s
Tufts	$x\in R^{150\times 8}$	15 s

**Table 4:** Three-way *n*-back task (0-, vs. 2- vs. 3-back) workload classification results on TUBerlin dataset. Mean accuracy and F1-Score are reported over 10 folds without DA (CE) and with DA by using different Subjects, Sessions, and Blocks as different domains at each experiment. Highest value in each column in bold. The overall highest value in the entire table is both bolded and underlined.

Model	CE (No DA)		Subject DA		Session DA		Blockwise-DA (Ours)	
	Acc	F1	Acc	F1	Acc	F1	Acc	F1
DeepConv	42.67%	**0.4053**	42.59% (−0.08%)	**0.4025** (−0.0028)	**42.69%** (0.02%)	**0.4057** (0.0004)	43.00% (0.33%)	0.4064 (0.0011)
EEGNet	**43.03%**	0.3968	42.36% (−0.67%)	0.3924 (−0.0044)	41.7% (−1.33%)	0.3942 (−0.0026)	42.44% (−0.59%)	0.4038 (0.0070)
MLPBiGRU	42.77%	0.3973	41.10% (−1.67%)	0.3914 (−0.0059)	42.63% (−0.41%)	0.3827 (−0.0146)	**43.35%** (0.58%)	0.3980 (0.0007)
MLPMixer	41.47%	0.3899	41.53% (0.06%)	0.3956 (0.0057)	42.28% (0.81%)	0.4013 (0.0114)	42.78% (**1.31%**)	**0.4112** (**0.0213**)

**Table 5:** Left-hand finger-tapping (LHT) vs. RHT vs foot-tapping (FT) classification results on FFT dataset. Mean accuracy and F1-Score are reported over 10 folds without DA (CE) and with DA by using different Subjects, Sessions, and Blocks as different domains. FFT was not collected in block design, but we view three consecutive trials as one block. Highest value in each column in bold. The overall highest value in the entire table is both bolded and underlined.

Model	CE (No DA)		Subject DA		Session DA		Blockwise-DA (Ours)	
	Acc	F1	Acc	F1	Acc	F1	Acc	F1
DeepConv	64.42%	0.5568	63.11% (−1.31%)	0.5542 (−0.0026)	62.98% (−1.53%)	0.5605 (0.0037)	64.64% (0.22%)	0.5710 (0.0142)
EEGNet	60.71%	0.5705	60.76% (0.04%)	0.6014 (0.0309)	60.93% (0.22%)	0.5995 (0.0290)	60.99% (0.28%)	0.6045 (0.0340)
MLPBiGRU	60.62%	0.5775	62.98% (2.36%)	0.6221 (0.0446)	62.76% (2.13%)	0.6262 (0.0487)	63.38% (**2.76%**)	0.6279 (**0.0505**)
MLPMixer	**64.76%**	**0.6519**	**64.58%** (−0.18%)	**0.6547** (0.0028)	**65.73%** (0.98%)	**0.6567** (0.0048)	**65.87%** (1.11%)	**0.6580** (0.0061)

**Table 6:** Binary *n*-back task workload (0- vs. 2-back) classification results on Tufts generic (cross subject) paradigm. Mean accuracy and F1-Score are reported over 17 folds without DA (CE) and with DA by using different Subjects, Sessions, and Blocks as different domains. Tufts dataset contains only one session for each subject. Highest value in each column in bold. The overall highest value in the entire table is both bolded and underlined.

Model	CE (No DA)		Subject DA		Session DA		Blockwise-DA (Ours)	
	Acc	F1	Acc	F1	Acc	F1	Acc	F1
DeepConv	63.75%	0.6245	61.96% (−1.79%)	0.6160 (−0.0085)	-	-	67.56% (3.80%)	0.6709 (0.0464)
EEGNet	62.08%	0.6153	65.37% (3.29%)	0.6509 (0.0356)	-	-	66.51% (**4.43%**)	0.6646 (**0.0493**)
MLPBiGRU	66.33%	0.6566	67.26% (0.92%)	0.6697 (0.0131)	-	-	67.40% (1.07%)	0.6726 (0.0160)
MLPMixer	67.28%	0.6726	**67.72%** (0.44%)	**0.6740** (0.0015)	-	-	**67.91%** (0.63%)	**0.6763** (0.0037)

**Table 7:** Paired *t*-test of F1 scores between CE baseline and proposed Blockwise-DA.

Dataset	Model	T-Statistic	*p*-Value
Tuberlin	DeepConv	0.2934	0.7759
	EEGNet	0.8418	0.4217
	MLPBiGRU	0.0987	0.9235
	MLPMixer	2.5608	0.0306
FFT	DeepConv	0.5292	0.6094
	EEGNet	0.8789	0.4023
	MLPBiGRU	2.6478	0.0266
	MLPMixer	0.735	0.481
Tufts	DeepConv	3.8882	0.0013
	EEGNet	4.052	0.0009
	MLPBiGRU	2.3861	0.0297
	MLPMixer	0.5032	0.6217

**Table 8:** Comparison of computation cost, in terms of Multiply-Accumulate (MAC) operations, at inference.

MACs	TUBerlin	FFT	Tufts
DeepConv [23]	956.3 K	6.03 M	3.12 M
EEGNet [22]	30.47 K	118.22 K	35.36 K
MLPBiGRU [15]	904.71 K	5.94 M	6.58 M
MLPMixer (ours)	409.76 K	2.4 M	2.37 M

## Data Availability

No new data were created or analyzed in this study.
